# Cell type-specific function of TRAF2 and TRAF3 in regulating type I IFN induction

**DOI:** 10.1186/s13578-018-0268-5

**Published:** 2019-01-03

**Authors:** Xiaoping Xie, Jin Jin, Lele Zhu, Zuliang Jie, Yanchuan Li, Baoyu Zhao, Xuhong Cheng, Pingwei Li, Shao-Cong Sun

**Affiliations:** 1 Department of Immunology, The University of Texas MD Anderson Cancer Center, 7455 Fannin Street, Box 902, Houston, TX 77030 USA; 2 Life Sciences Institute, Zhejiang University, Hangzhou, 310058 China; 3 Department of Biochemistry and Biophysics, Texas A&M University, College Station, TX USA; 4 The University of Texas Graduate School of Biomedical Sciences at Houston, Houston, TX 77030 USA

**Keywords:** TRAF2, TRAF3, Type I interferon, Antiviral immunity

## Abstract

**Background:**

TRAF3 is known as a central mediator of type I interferon (IFN) induction by various pattern recognition receptors, but the in vivo function of TRAF3 in host defense against viral infection is poorly defined due to the lack of a viable mouse model.

**Results:**

Here we show that mice carrying conditional deletion of TRAF3 in myeloid cells or dendritic cells do not have a significant defect in host defense against vesicular stomatitis virus (VSV) infection. However, whole-body inducible deletion of TRAF3 renders mice more sensitive to VSV infection. Consistently, TRAF3 was essential for type I IFN induction in mouse embryonic fibroblasts (MEFs) but not in macrophages. In dendritic cells, TRAF3 was required for type I IFN induction by TLR ligands but not by viruses. We further show that the IFN-regulating function is not unique to TRAF3, since TRAF2 is an essential mediator of type I IFN induction in several cell types, including macrophages, DCs, and MEFs.

**Conclusions:**

These findings suggest that both TRAF2 and TRAF3 play a crucial role in type I IFN induction, but their functions are cell type- and stimulus-specific.

## Background

Type I interferon (IFN) plays a crucial role in innate immunity against viral infections [1, 2]. Induction of type I IFNs is typically mediated by pattern-recognition receptors (PRRs), including toll-like receptors (TLRs), RIG-I-like receptors (RLRs), NOD-like receptors, and the cytosolic GAMP synthase (cGAS), which detect various molecular patterns associated with viral genomes or replication products [2, 3]. In response to ligand stimulation, the PRRs transduce signals via specific signaling adaptors. TIR-domain-containing adapter-inducing interferon-β (TRIF) mediates type I IFN induction by TLR3 and TLR4 upon stimulation by double-stranded RNA and lipopolysaccharide (LPS), respectively. Mitochondrial antiviral-signaling protein (MAVS, also known as VISA and IPS-1) serves as an adaptor of RLRs, whereas stimulator of interferon gene (STING) mediates type I IFN induction by cGAS and other cytoplasmic DNA sensors [2, 4]. A common downstream target of the different PRR pathways is the protein kinase TBK1, which upon activation phosphorylates and activates the transcription factor interferon regulatory factor 3 (IRF3) [4, 5]. Phosphorylated IRF3 forms a dimer and translocates to the nucleus to participate in the induction of type I IFN genes.

Activation of TBK1 and IRF3 by the PRRs also requires members of the tumor necrosis factor receptor (TNFR)-associated factors (TRAFs) family [3, 6]. TRAFs are adaptor proteins or E3 ubiquitin ligases that transduce signals from TNFR superfamily as well as other immune receptors, including the PRRs [7]. While the typical functions of TRAFs include activation of NF-kB and MAP kinase signaling pathways, TRAF3 has been shown to mediate activation of IRF3 and implicated as a common signaling adaptor for type I IFN induction [2, 8, 9]. However, whether TRAF3 is a specific TRAF member that mediates type I IFN induction is in debate, since in some systems other TRAF members are also involved in the type I IFN induction [10, 11]. The in vivo function of TRAF3 in mediating antiviral host defense is also poorly defined, since current studies have been largely relied on in vitro systems using immortalized mouse embryonic fibroblasts (MEFs) or other cell lines.

To study the physiological function of TRAF3 in antiviral innate immunity, we employed TRAF3 conditional knockout (KO) mice carrying TRAF3 deficiencies in different immune cell types or with inducible deletion of TRAF3 in adult mice. We obtained genetic evidence that whole-body inducible deletion of TRAF3 in adult mice attenuated their host defense against vesicular stomatitis virus (VSV) infection. Surprisingly, however, TRAF3 was dispensable in both myeloid cells and dendritic cells for this innate immune function. Using primary cells, we further demonstrated that the function of TRAF3 in mediating type I IFN induction is cell type- and stimulus-specific. Moreover, this innate immune function is not unique for TRAF3, since TRAF2 is equally important for type I IFN induction. The results provide new insight into the physiological function of TRAF proteins in mediating antiviral innate immunity.

## Results

### In vivo function of TRAF3 in host defense against viral infection

**Fig. 1:**
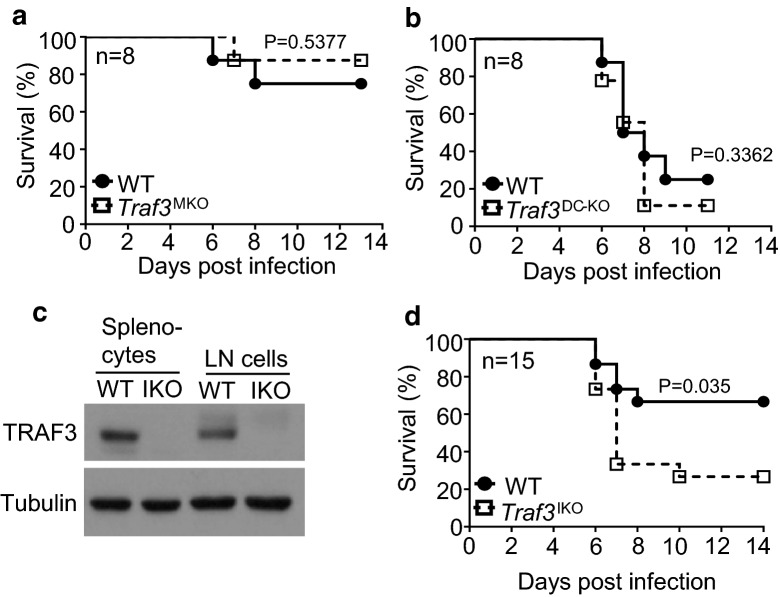
Survival of *Traf3* conditional KO mice following VSV infection. **a** Age-matched (6–8 weeks old) WT and *Traf3*^MKO^ mice were infected i.v. with VSV (1 × 10^7^ PFU per mouse). Data are presented as 8 animals/group. **b** *Traf3*^DC-KO^ and WT littermate control mice were infected i.v. with VSV (2 × 10^7^ PFU per mouse). Survival was recorded and presented as 8 mice per group. **c** Immunoblot analysis of the indicated proteins in the extracts of splenocytes or LN cells derived from Tamoxifen-treated WT and *TRAF3*^IKO^ mice. **d** WT and *TRAF3*^IKO^ mice (n = 15) were infected with VSV (2 × 10^7^ PFU per mouse) via tail vein. Data representative of two independent experiments, and statistical significance was determined by log-rank and Gehan-Wilcoxon tests. p < 0.05 indicates significantly different

Germline deletion of TRAF3 causes postnatal lethality, which has hampered studies to examine in vivo functions of TRAF3 [12]. To study the in vivo role of TRAF3 in regulating antiviral innate immunity, we generated myeloid cell-conditional *Traf3* KO (Traf3^fl/fl^*Lyz2*-Cre; hereafter called *Traf3*^MKO^) mice by crossing *Traf3*-flox mice [13] with *Lyz2*-Cre [14]. We then challenged these mice with VSV, a frequently used model for studying host defense against RNA virus infections [15]. VSV is known to induce type I IFN induction via the cytoplasmic RNA-responsive PRR RIG-I [15]. Surprisingly, TRAF3 deletion in myeloid cells did not sensitize mice to VSV infection, since the *Traf3*^MKO^ and wild-type control mice displayed comparable survival rate following intravenous (i.v.) infection with VSV (Fig. 1a). Since dendritic cells (DCs) also play a crucial role in regulating antiviral immunity, we next examined the in vivo role of TRAF3 in DCs by generating DC-conditional Traf3 KO (*Traf3*^fl/fl^*Cd11c*-Cre; here after called *Traf3*^DC-KO^) mice. Similar to the *Traf3*^MKO^ mice, the *Traf3*^DC-KO^ mice did not show enhanced sensitivity to VSV infection compared to their age-matched wild-type control mice (Fig. 1b). These results suggest that TRAF3 is dispensable in innate immune cells for host defense against VSV infections.

To further investigate the antiviral innate immune function of TRAF3, we employed an inducible KO system allowing TRAF3 deletion in different cell types in adult mice. We crossed the *Traf3*-flox mice with transgenic mice expressing a tamoxifen-inducible Cre recombinase, ER-Cre [16]. We then injected the *Traf3*^fl/fl^ER^Cre/+^ and control *Traf3*^fl/fl^ mice with tamoxifen to generate the *Traf3* inducible KO (*Traf3*^IKO^) and wild-type control mice. Immunoblot analysis using splenocytes and lymph node cells revealed efficient deletion of TRAF3 in the *Traf3*^IKO^ mice (Fig. 1c). Importantly, the whole-body deletion of TRAF3 in adult mice significantly attenuated the host defense against VSV infection (Fig. 1d). These results suggest that TRAF3 plays an important role in mediating antiviral immunity, but it may have cell type-specific functions.

### TRAF3 is required for type I IFN induction in MEFs but not in macrophages

**Fig. 2:**
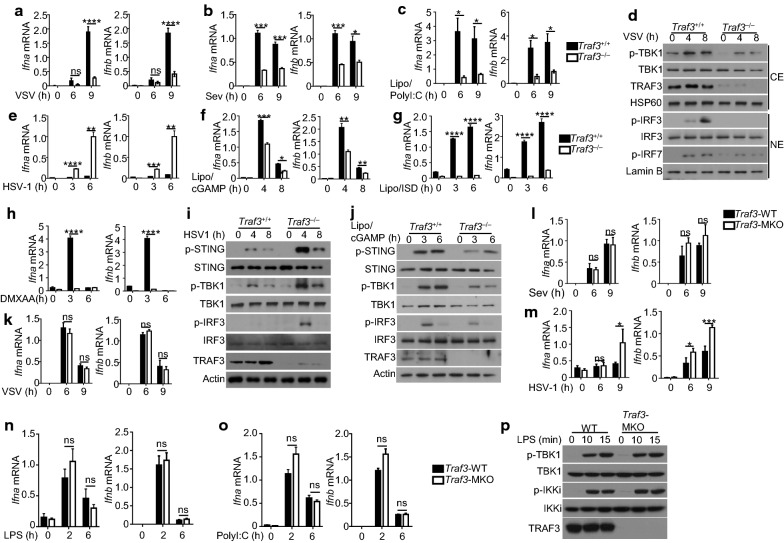
Cell type-specific function of TRAF3 in regulating type I IFN induction. qRT-PCR analysis of *Ifna* and *Ifnb* mRNA expression in WT and *Traf3*^–/–^ primary MEFs infected with VSV (**a**) or SeV (**b**) or treated with transfected with poly(I:C) (**c**) for the indicated times. **d** Immunoblot analysis of the indicated phosphorylated (P−) and total proteins in the cytoplasmic (CE) and nuclear (NE) extracts of WT and *Traf3*^–/–^ primary MEFs infected with VSV (M.O.I = 10) for the indicated time period. qRT-PCR analysis of relative Ifna and Ifnb mRNA amounts in WT and *Traf3*^–/–^ MEF cells infected with HSV-1 (**e**) or treated with lipofectamine-transfected cGAMP (**f**), lipofectamine-transfected ISD (**g**), or stimulated with DMXAA (**h**) for the indicated time periods. **i**, **j** Immunoblot analysis of the indicated proteins in whole-cell lysates of WT or *Traf3*^–/–^ MEFs infected with HSV-1 (**i**) or lipofectamine-transfeted cGAMP (**j**) for the indicated time periods. qRT-PCR analysis of *Ifna* and *Ifnb* mRNA relative level in BMDMs derived from WT or *Traf3*^MKO^ mice infected with VSV (**k**), SeV (**l**), HSV-1 (**m**) or stimulated with LPS (**n**) or poly(I:C) (**o**). **p** Immunoblot analysis of the indicated proteins in whole-cell lysates of WT or *Traf3*^MKO^ BMDMs stimulated with LPS for the indicated time periods. All qRT-PCR data are presented as fold relative to the amount of an internal control, *Actb*. Data are representative of three to four independent experiments, and statistical analyses are presented as mean ± SEM. *p < 0.05, **p < 0.01, ***p < 0.001 and ****p < 0.0001. *ns* nonsignificant

To better understand the cell type-specific functions of TRAF3, we prepared primary MEFs as well as innate immune cells from TRAF3-deficient or wild-type control mice. In agreement with previous studies [8, 9], TRAF3 deficiency in primary MEFs severely attenuated the *Ifna* and *Ifnb* induction by the RNA viruses VSV and Sendai virus (SeV) (Fig. 2a, b). In addition, the TRAF3-deficient MEFs were also defective in type I IFN induction by transfected polyI:C known to mimic RNA viruses and stimulate the RIG-I signaling pathway (Fig. 2c). Consistently, parallel immunoblot assays revealed reduced phosphorylation of TBK1 and its target transcription factor IRF3 in TRAF3-deficient MEFs stimulated by VSV (Fig. 2d). Thus, TRAF3 is required for type I IFN induction by the RIG-I pathway in primary MEFs.

A recent study suggests that TRAF3 negatively regulates DNA virus-induced type I IFN induction with a mechanism that involves STING activation by the TRAF3-controlled kinase NIK [17]. Consistent with this report, we found that the TRAF3-deficient MEFs had greatly enhanced type I IFN induction by a DNA virus, herpes simplex virus 1 (HSV-1) (Fig. 2e). Surprisingly, however, the TRAF3 deficiency did not promote, but rather significantly attenuated, the induction of type I IFN induction by non-viral STING agonists, cGAMP (Fig. 2f), ISD (Fig. 2g) or DMXAA (Fig. 2h). Consistently, while TRAF3 deficiency promoted the activation of STING/TBK1/IRF3 signaling pathways by HSV-1 (Fig. 2i), it attenuated these signaling events induced by cGAMP (Fig. 2j). These results suggest that TRAF3 may play different roles in regulating STING activation and upstream signaling steps in the DNA-sensing pathway.

To examine the role of TRAF3 in innate immune cells, we prepared primary bone barrow derived macrophages (BMDMs) from *Traf3*^MKO^ and wild-type control mice. In contrast to the results obtained with MEFs, TRAF3 deletion in BMDMs had no effect on the induction of *Ifna* and *Ifnb* genes by VSV or SeV (Fig. 2k, l). On the other hand, as seen in MEFs, the TRAF3 deficiency in macrophages promoted type I IFN induction by the DNA virus HSV (Fig. 2m). Furthermore, TRAF3 was completely dispensable for the induction of *Ifna* and *Ifnb* by the TLR ligands LPS and polyI:C (Fig. 2n, o). Consistently, loss of TRAF3 in macrophages did not affect LPS-stimulated phosphorylation of TBK1 or its homolog IKKi (Fig. 2p). These results suggest cell type-specific functions of TRAF3 in mediating type I IFN induction.

### Inducer-specific function of TRAF3 in DCs

**Fig. 3:**
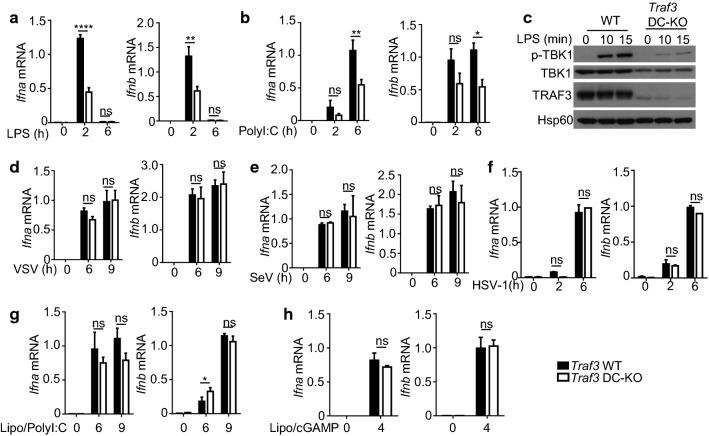
Stimulus-specific function of TRAF3 in regulating type I IFN induction in BMDCs. **a**, **b** qRT-PCR analysis of *Ifna* and *Ifnb* expression in BMDCs derived from WT or *Traf3*^DC-KO^ mice stimulated with LPS (**a**) or poly(I:C) (**b**). **c** Immunoblot analysis of the indicated proteins in whole-cell lysates of WT or *Traf3*^DC-KO^ BMDCs stimulated with LPS for the indicated time points. qRT-PCR analysis of *Ifna* and *Ifnb* mRNA expression in WT and *Traf3*^DC-KO^ BMDCs infected with VSV (**d**), Sev (**e**) or HSV-1 (**f**) or treated with lipofectamine-transfected poly(I:C) (**g**) or cGAMP (**h**) for the indicated time periods. Data are representative of three independent experiments, and statistical analyses are presented as mean ± SEM. *p < 0.05, **p < 0.01, ***p < 0.001 and ****p < 0.0001. *ns* nonsignificant

DCs also serve as an important innate immune cell type mediating host defense against viral infections. We thus examined the role of TRAF3 in regulating type I IFN induction in primary bone marrow derived DCs (BMDCs). Compared to the wild-type BMDCs, the TRAF3-deficient BMDCs had a significant reduction in type I IFN gene induction by both LPS and polyI:C (Fig. 3a, b). Consistently, the TRAF3-deficient DCs also had impaired induction of TBK1 phosphorylation by LPS (Fig. 3c). Interestingly, however, TRAF3 was completely dispensable for induction of *Ifna* and *Ifnb* by the RNA viruses VSV and SeV (Fig. 3d, e). Similarly, TRAF3 deletion in DCs had no significant effect on type I IFN induction by the DNA virus HSV (Fig. 3f). To further confirm these results, we also directly stimulated the RIG-I and STING pathways using transfected polyI:C and cGAMP, respectively. Once again, induction of type I IFN gene expression by these inducers did not require TRAF3 in DCs (Fig. 3g, h). Thus, the function of TRAF3 in mediating type I IFN induction is dependent on inducers as well as cell types.

### TRAF2 is an essential mediator of type I IFN induction in multiple cell types

**Fig. 4:**
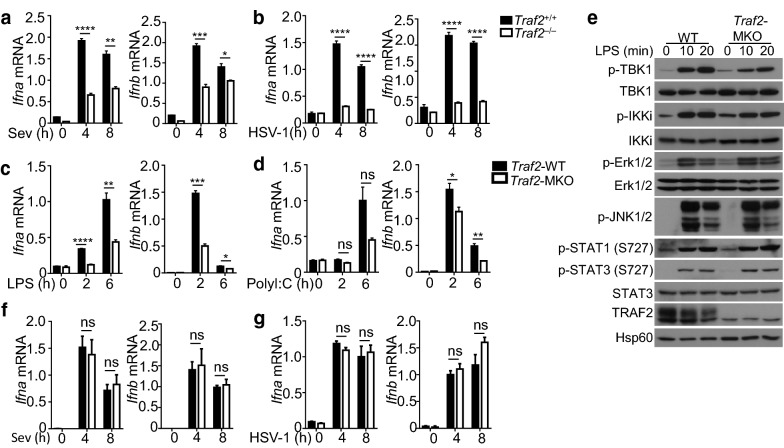
Role of TRAF2 in regulating type I IFN Induction. qRT-PCR analysis of *Ifna* and *Ifnb* expression in WT or *TRAF2*^–/–^ primary MEFs infected with Sev (**a**) or HSV-1 (**b**). **c**, **d** qRT-PCR analysis of *Ifna* and *Ifnb* expression in BMDMs derived from WT or *Traf2*^MKO^ mice stimulated with LPS (**c**) or poly(I:C) (**d**). **e** Immunoblot analysis of the indicated proteins in whole-cell lysates of WT or *TRAF2*^MKO^ BMDMs stimulated with LPS for the indicated time points. **f**, **g** qRT-PCR analysis of *Ifna* and *Ifnb* mRNA expression in WT and *Traf2*^MKO^ BMDM cells infected with Sev (**f**) or HSV-1 (**g**). Data are representative of three independent experiments, and statistical analyses are presented as mean ± SEM. *p < 0.05, **p < 0.01, ***p < 0.001 and ****p < 0.0001. *ns* nonsignificant

TRAF3 has been frequently cited in the literature as a central mediator of antiviral innate immunity; however, it is unclear whether this function is unique for TRAF3 or also for other TRAF members. To address this question, we examined the role of TRAF2 by generating primary cells from TRAF2-conditional KO and wild-type control mice. Interestingly, like TRAF3, TRAF2 was essential for type I IFN induction by the RNA virus SeV in primary MEFs (Fig. 4a). This finding was intriguing, since it suggests that deletion of either TRAF2 or TRAF3 in MEFs blocks type I IFN induction, thus suggesting non-redundant functions of TRAF2 and TRAF3. Remarkably, TRAF2 was also required for type I IFN induction by the DNA virus HSV-1 (Fig. 4b), which was in sharp contrast to the negative role of TRAF3 in mediating the DNA-sensing pathway (Fig. 2e). These results suggest that TRAF2 has both similar and different functions from TRAF3 in the regulation of type I IFN induction.

We next examined the role of TRAF2 in regulating type I IFN induction by TRIF-dependent TLRs, TLR3 and TLR4, using macrophages. BMDMs prepared for the *Traf2*^MKO^ mice had a severe defect in LPS-stimulated *Ifna* and *Ifnb* expression and also a significant reduction in polyI:C-stimulated *Ifnb* expression (Fig. 4c, d). Consistently, the TRAF2 deficiency attenuated, although did not completely block, LPS-stimulated TBK1 phosphorylation (Fig. 4e). TRAF2 deficiency did not appreciablely alter the activation of IKKi or other signaling factors, suggesting the involvement of TRAF2 in regulating the TBK1 signaling axis. Parallel studies using the RNA virus SeV and the DNA virus HSV-1 did not reveal significant differences in *Ifna* and *Ifnb* induction between the *Traf2*^MKO^ and wild-type control macrophages (Fig. 4f, g).

**Fig. 5:**
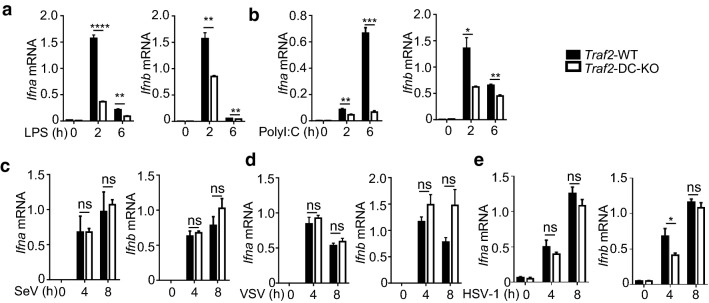
Stimulus-specific function of TRAF2 in regulating type I IFN induction in DCs. **a**, **b** qRT-PCR analysis of *Ifna* and *Ifnb* mRNA expression in WT and *Traf2*^DC-KO^ BMDCs stimulated with LPS (**a**) or poly(I:C) (**b**) or infected with Sev (**c**), VSV (**d**) or HSV-1 (**e**). Data are representative of three independent experiments, and statistical analyses are presented as mean ± SEM. *p < 0.05, **p < 0.01, ***p < 0.001 and ****p < 0.0001. *ns* nonsignificant

To investigate the role of TRAF2 in DCs, we generated DC-conditional TRAF2 KO (Traf2^DC-KO^) mice. BMDCs prepared from the Traf2^DC-KO^ mice displayed a severe defect in type I IFN induction by LPS and polyI:C (Fig. 5a, b). In contrast to the TLR pathways, induction of type I IFNs by the RNA viruses, SeV and VSV, and the DNA virus HSV-1 was not affected by the TRAF2 deficiency in DCs (Fig. 5c–e). Collectively, these results suggest that like TRAF3, TRAF2 has an essential role in mediating type I IFN induction in an inducer-specific manner.

## Discussion

The results presented in the present study provide genetic evidence that TRAF3 mediates antiviral host defense in mice. Our data support the previous reports that TRAF3 plays a crucial role in regulating type I IFN induction [8, 9]. However, by employing different types of primary cells as well as *Traf3* conditional KO mice, we demonstrated that this function of TRAF3 was cell type- and stimulus-specific (Table 1). Surprisingly, deletion of TRAF3 in either myeloid cells or dendritic cells had no effect on host defense against VSV infection, but whole-body inducible deletion of TRAF3 impaired this innate immune function. Consistent with these in vivo results, cell culture studies revealed that TRAF3 was dispensable for type I IFN induction by both TLR ligands and viruses in macrophages. Furthermore, TRAF3 deletion in DCs had no effect on virus-induced type I IFN induction, although it inhibited IFN induction by TLR3 and TLR4 ligands. In contrast, TRAF3 was essential for IFN induction by RNA viruses in MEFs. These results suggest that TRAF3 is dispensable for antiviral responses in innate immune cells but may play an essential role in other cell types, such as fibroblasts.

**Table 1:** Cell-type specific effect of TRAF3 deficiency on type I IFN induction

Pathways	Inducers	Cell types		
		MEFs	BMDM	BMDC
RIG-I pathway	VSV	Inhibited	No effect	No effect
	Sev	Inhibited	No effect	No effect
	Lipo/PolyI:C	Inhibited	NA	No effect
STING pathway	HSV1	Upregulated	Upregulated	No effect
	Lipo/cGAMP	Inhibited	NA	No effect
	Lipo/ISD	Inhibited	NA	NA
	DMXAA	Inhibited	NA	NA
TRIF pathway	LPS	NA	No effect	Inhibited
	PolyI:C	NA	No effect	Inhibited

*NA* not analyzed

Our finding that TRAF3 is dispensable in myeloid cells for innate immunity against VSV infection is in agreement with a previous study that myeloid cell-specific deletion of TRAF3 has no effect on LPS-induced IFNβ production in vivo [18]. However, in contrast to our present finding that TRAF3 is dispensable for LPS-stimulated type IFN expression in macrophages, this prior study suggests that TRAF3 is required for in vitro type I IFN induction by LPS in macrophages. The reason for this discrepancy is unclear, but it could be due to the use of two different *Traf3*-flox mouse strains or differences in experimental conditions. Nevertheless, we found that TRAF3 deletion in macrophages also did not affect type I IFN induction by another TLR ligand, polyI:C. Future studies will further study the role of TRAF3 and other TRAF members in TLR-stimulated type I IFN induction.

Although TRAF3 has been implicated as a specific antiviral signaling adaptor, our data suggest that TRAF2 is equally important for mediating type I IFN induction. In fact, TRAF2 displayed an essential role in mediating type I IFN induction in several cell types, including MEFs, macrophages, and DCs (Table 2). In macrophages and DCs, TRAF2 appeared to be more important for the TRIF-dependent TLR pathways, since TRAF2 deletion in these cells inhibited type I IFN induction by TLR3 and TLR4 ligands but not by RNA or DNA viruses. The molecular mechanism underlying the cell type- and inducer-specific functions of TRAF2 and TRAF3 is currently unclear, but it may involve functional redundancy with other TRAF members. However, it is remarkable that TRAF2 and TRAF3 both had non-redundant roles in type I IFN induction in the same cell types, including MEFs and DCs, since deletion of either TRAF2 or TRAF3 impaired the induction of type I IFNs by the same types of inducers. This finding suggests that TRAF2 and TRAF3 may function cooperatively, as seen in the regulation of the noncanonical NF-kB kinase NIK [19], or at different steps of the IFN induction pathways.

**Table 2:** Cell-type specific effect of TRAF2 deficiency on type I IFN induction

Pathways	Inducers	Cell types		
		MEFs	BMDM	BMDC
RIG-I pathway	VSV	NA	NA	No effect
	Sev	Inhibited	No effect	No effect
STING pathway	HSV1	Inhibited	No effect	No effect
TRIF pathway	LPS	NA	Inhibited	Inhibited
	PolyI:C	NA	Inhibited	Inhibited

*NA* not analyzed

A recent study suggests that TRAF3 negatively regulates type I IFN induction by DNA viruses, such as HSV-1, through controlling the noncanonical NF-κB inducing kinase NIK [17]. Accumulation of NIK due to TRAF3 deficiency promotes STING activation and induction of type I IFNs [17]. In support of this report, we found that TRAF3 deletion in MEFs and macrophages promotes HSV-1-stimulated expression of *Ifna* and *Ifnb* genes. However, the mechanism by which TRAF3 regulates STING pathway appears to be complex. First, while TRAF3 deficiency promoted type I IFN induction by HSV-1, it inhibited type I IFN induction by the STING stimulators cGAMP, ISD, and DMXAA. These results suggest that TRAF3 may have dual functions in DNA-stimulated type I IFN production. It is possible that TRAF3 may positively regulate STING activation of TBK1 or IRF3 and negatively regulates an upstream step in the DNA-sensing pathway. Second, TRAF2 deficiency, which also causes NIK accumulation [20], did not promote type I IFN induction by HSV-1, which argues for the involvement of a NIK-independent mechanism. In fact, deletion of TRAF2 in MEFs largely blocked the induction of type I IFN genes by HSV-1. These results, along with the finding that TRAF3 deficiency blocks cGAMP-induced type I IFN induction, further suggest a positive role for TRAF2 and TRAF3 in regulating STING-mediated signaling. HSV-1 has evolved multiple strategies to evade host antiviral responses. Some HSV-1-encoded gene products have been identified inhibitors targeting upstream steps of the STING/TBK1/IRF3 pathway. For example, the HSV-1 tegument protein UL41 inhibits type I IFN induction by selectively degrading the mRNA of cGAS, a key DNA sensor mediating cGAMP synthesis and STING activation by DNA viruses [21]. Another HSV-1 tegument protein, UL37, deamidates mouse cGAS, impairing its ability to catalyze cGAMP synthesis [22]. Moreover, an HSV-1 virulence factor, γ134.5, inhibits STING activation by interfering the translocation of STING from endoplasmic reticulum to Golgi apparatus [23]. Future studies will examine whether TRAF3 plays a role in regulating the interplay between these HSV-1 components and host STING signaling pathway.

## Conclusions

The findings of the present study suggest that in addition to TRAF3, TRAF2 is a crucial mediator of type I IFN induction and that the function of TRAF2 and TRAF3 is cell type- and stimulus-specific.

## Materials and methods

### Mice

Traf2-flox and Traf3-flox mice were provided by Dr. Robert Brink (Garvan Institute of Medical Research) [13]. Traf3-flox mice were further crossed with EIIa-Cre mice, lysozyme 2-Cre (Lyz2-Cre) mice or CD11c-Cre mice to produce age-matched *Traf3* germ line knockout mice (termed *Traf3*^–/–^), Traf3^fl/fl^ lyz2^Cre/+^ (termed *Traf3*^MKO^), Traf3^fl/fl^ CD11c^Cre^ (termed *Traf3*^DC-KO^) mice and Traf3^fl/fl^ (termed *Traf3*^+/+^) mice. Also, *Traf2*-flox mice were crossed with EIIa-Cre mice, Lyz2-Cre mice or CD11c-Cre mice to produce age-matched *Traf2* germ line knockout mice (termed *Traf2*^–/–^), *Traf2*^fl/fl^ lyz2^Cre/+^ (termed *Traf2*^MKO^), Traf2^fl/fl^ CD11c^Cre^ (termed *Traf2*^DC-KO^) mice and *Traf2*^fl/fl^ (termed *Traf2*^+/+^) mice. *Traf3*-flox mice were also crossed with CAGG-Cre-ER mice to generate age matched tamoxifen inducible Traf3^fl/fl^ ER^Cre/+^ mice and Traf3^fl/fl^ mice; these mice were then injected intraperitoneally with 100 μl tamoxifen (20 mg/ml in corn oil solution) for a total of 5 consecutive days with 24 h intervals, creating *Traf3* inducible KO (*Traf3*^IKO^) and wild-type control mice. Seven days after the last tamoxifen injection, the mice were used for viral infection experiments. All mouse strains were in C57BL/6 background. The mice were maintained in specific pathogen-free facility of The University of Texas MD Anderson Cancer Center, and all animal experiments were conducted in accordance with protocols approved by the Institutional Animal Care and Use Committee.

### Viruses, antibodies, and reagents

VSV (Indiana strain) was provided by Glen Barber (University of Miami), and a VSV variant harboring a point mutation in the M gene (AV1) was provided by John Bell (Ottawa Hospital Research Institute) [24]. Vero cells were used to propagate and determine the titers of HSV-1 (KOS), and BHK21 cells were used for generating VSV WT and VSV-AV1 Mutant viruses. Antibodies for STING, p-STING (Ser365), TBK1, p-TBK1, p-IKKe, IKKi, p52, p-IRF7, p-Erk, p-STAT3 (Ser727), p-STAT1 (S727), STAT3 and Tubulin were purchased from Cell Signaling Technology Inc. Antibodies for Hsp60, TRAF2, TRAF3, Erk, LaminB and IRF3 were purchased from Santa Cruz Biotechnology. p-IRF3 was purchased from ThermoFisher Scientific. LPS (derived from *Escherichia coli* strain 0127:B8) were from Sigma Aldrich. Poly (I:C) was from Amersham. cGAMP was synthesized as previously described [25]. Recombinant murine M-CSF and GM-CSF were from Peprotech. Murine STING ligand DMXAA and ISD naked were purchased from InvivoGen.

### Cell preparation and stimulation

Bone marrows were prepared from the femurs of adult mice and cultured in a M-CSF conditional medium for BMDM differentiation. BMDCs were generated by cultivating bone marrow cells in RPMI medium supplemented with GM-CSF (10 ng/ml) for 7 days, and the differentiated BMDCs were stained with Pacific blue–conjugated anti-CD11c and purified by flow cytometric cell sorting as described [26]. To prepare primary MEFs, we bred heterozygous mice for obtaining the KO and WT embryos from the same pregnant female mice. The MEFs, BMDMs, and BMDCs were starved overnight in medium supplemented with 0.5% FCS before being stimulated with LPS (1 μg/ml for immunoblot experiments and 100 ng/ml for cytokine induction experiments), poly(I:C) (20 μg/ml), DMXAA (50 μg/ml), Lipofectamine-transfected poly(I:C) (20 μg/ml), cGAMP (10 μg/ml) or ISD (1 μg/ml) for the indicated times. Total and subcellular extracts were prepared for immunoblot assays, and total RNA was prepared for QPCR assays.

### Quantitative RT-PCR

Total RNA was isolated using Trizol reagent (Molecular Research Center, Inc.) and subjected to cDNA synthesis using RNase H-reverse transcriptase (Invitrogen) and oligo (dT) primers. qRT-PCR was performed in triplicates, using iCycler Sequence Detection System (Bio-Rad) and iQTM SYBR Green Supermix (Bio-Rad). The expression of individual genes was calculated by a standard curve method and normalized to the expression of *Actb*. The mouse gene-specific PCR primers are used as follows: *Actb* forward primer: 5′CGTGAAAAGATGACCCAGATCA, *Actb* reverse primer: 5′CACAGCCTGGATGGCTACGT3′; *Ifnb* forward primer: 5′AGCTCCAAGAAAGGACGAACAT3′, *Ifnb* reverse primer: 5′GCCCTGTAGGTGAGGTTGATCT3′; and *Ifna* forward primer: 5′TGACCTCAAAGCCTGTGTGATG3′, *Ifna* reverse primer: 5′AAGTATTTCCTCACAGCCAGCAG3′.

### Immunoblot

Whole-cell lysates or subcellular extracts were prepared and subjected to immunoblot assays as described [27, 28]. The samples were resolved by 8.25% SDS-PAGE. After electrophoresis, separated proteins were transferred onto polyvinylidene difluoride membrane (Millipore). For IB assays, the polyvinylidene difluoride membrane was blocked with 5% non-fat milk. After incubation with specific primary antibody, horseradish peroxidase-conjugated secondary antibody was applied. The positive immune reactive signal was detected by ECL (Amersham Biosciences).

### Viral infection

For gene induction and signaling analysis, MEFs were seeded into 12-well plates (5 × 10^5^ cells per well) and infected with VSV-AV1 mutant, HSV-1 (KOS) or Sev strains in serum-free medium for 1 h. The cells were washed once and cultured in growth medium for the indicated time periods and then collected for immunoblot or QPCR assays. For mouse infection, the indicated age-matched (6–8 weeks’ old) *Traf3* conditional KO or WT control mice were housed in a biosafety level 2 facility and infected i.v. with VSV (2 × 10^7^ PFU per mouse in 200 μl). The infected mice were monitored for survival up to 14 days.

### Statistical analysis

Prism software was used for two-tailed unpaired t-tests and data are presented as mean ± SEM. Log-rank and Gehan-Wilcoxon tests were performed for survival curves. p-values < 0.05 and 0.01 are considered significant and very significant, respectively.

## Abbreviations

IFN: interferon
VSV: vascular stomatitis virus
SeV: Sendai virus
HSV-1: herpes simplex virus 1
MEF: mouse embryonic fibroblast
PRR: pattern-recognition receptor
TLR: toll-like receptor
RLR: RIG-I-like receptor
cGAS: cytosolic GAMP synthase
TRIF: TIR-domain-containing adapter-inducing interferon-β
LPS: lipopolysaccharide
MAVS: mitochondrial antiviral-signaling protein
STING: stimulator of interferon gene
IRF3: interferon regulatory factor 3
TNFR: tumor necrosis factor receptor
TRAF: TNFR-associated factor
KO: knockout
DC: dendritic cell
BMDC: bone marrow derived DC
BMDM: bone marrow derived macrophage
